# The journey to establishing an IT-infrastructure within the German Biobank Alliance

**DOI:** 10.1371/journal.pone.0257632

**Published:** 2021-09-22

**Authors:** Christina Schüttler, Hans-Ulrich Prokosch, Michael Hummel, Martin Lablans, Björn Kroll, Cäcilia Engels, on behalf of the German Biobank Alliance IT development team

**Affiliations:** 1 Medical Informatics, Friedrich-Alexander-Universität Erlangen-Nürnberg, Erlangen, Germany; 2 German Biobank Node, Charité –Universitätsmedizin Berlin, Berlin, Germany; 3 Federated Information Systems, German Cancer Research Center, Heidelberg, Germany; 4 University Medical Center Mannheim, Mannheim, Germany; 5 IT Center for Clinical Research, University of Lübeck, Lübeck, Germany; Rutgers Robert Wood Johnson Medical School, UNITED STATES

## Abstract

**Background:**

Biobanks ensure the long-term storage and accessibility of biospecimens and corresponding data sets. Thus, they form the foundation for many research projects which may contribute to improving medical care. With the establishment of the German Biobank Node and Alliance, expertise in biobanking is bundled and strengthened. An important component within this research infrastructure is the set-up of an information technology (IT) network for allowing feasibility requests across individual biobanks.

**Objective:**

We aim to describe relevant aspects that have shaped the journey to interconnect biobanks, to enhance their visibility within the research-community, to harmonize data, and to enable feasibility searches to support access to available data and biosamples.

**Methods:**

To achieve this task, we resorted to a wide variety of methods: we ran a requirement analysis, decided on the mode of operation for the federated team of IT-developers and on the development approach itself, took related national and international initiatives into account, and concluded with evaluations of the developed software artefacts and the operation of the entire chain of applications.

**Results:**

We drew an IT framework including all heterogeneous data aspects derived from our requirement analysis and developed a comprehensive IT infrastructure. The successful implementation benefited from a smooth interaction of a federated IT team distributed across all participating sites that was even able to manage a major technology change mid-project. Authentication and project management services from associated partners could be integrated and the graphic user interface for an intuitive search tool for biospecimens was designed iteratively. The developed code is open source to ensure sustainability and the local implementation is concluded and functioning. The evaluation of the components was positive.

**Conclusions:**

The entire project had given ample opportunity for challenges, predictable and unpredictable—from the mode of operation to changing some of the initial ideas. We learned our lessons concerning personnel, budget planning and technical as well as manual monitoring as well as some requirements arising only during the process of the project. Nevertheless, we can here report a success story of a network infrastructure, highly agile and much easier in local installation than initially anticipated.

## Introduction

Biobanks are an indispensable instrument of disease- and patient-oriented biomedical research. They ensure the long-term storage and accessibility of biosamples and the corresponding data sets. Thus, biobanks provide an important basis for conducting research which boost the development of future diagnostics and precision therapy [1, 2]. Personalized precision medicine largely relies on large-scale high-throughput analyses of high-quality and well-described disease-specific patient samples and holds great opportunities to advance and improve modern approaches to healthcare [3, 4]. Biobanks are hubs for the acquisition, processing, and storage of biospecimens, providing both high quality tissue and fluid samples and access to services that process them into derivatives such as serum, plasma, DNA, and RNA. This, in combination with linked disease and socio-demographic data of the donor, provides a rich source for the research community [5].

In an effort to make biosamples and associated data accessible, the German Federal Ministry of Education and Research funded the establishment of a central organization for coordination and steering national biobank activities, the German Biobank Node (GBN) [6–8], which also acts as a hub for the European biobank infrastructure BBMRI-ERIC (Biobanking and Biomolecular Resources Research Infrastructure–European Research Infrastructure Consortium) [9]. One central pillar for this next generation biobanking is the construction of a sophisticated information technology (IT) network. This is a crucial factor to enable higher visibility and efficient access to biosamples and data in order to compile large, multicentric sample collections for research at national and international level. To this end, the German Biobank Alliance (GBA) was formed under the umbrella of GBN.

Since the local IT situation for both, biobanking IT and the general hospital information systems, is heterogeneous throughout Germany, the primary goal of GBA was the gradual IT connection of biobanks to a central network infrastructure by providing appropriate interfaces and locally missing components. The infrastructure is designed to cover two basic aspects. On the one hand, a legally compliant IT framework had to be created to facilitate the allocation of biosamples and associated data via a central IT platform (feasibility queries). On the other hand, this particular platform had to be developed.

The objective of this paper is to outline our approach of establishing and implementing this IT infrastructure. Special attention will be paid to the exceptional situation that the development team was scattered over several locations in Germany. Furthermore, it discusses the challenges, the lessons learned, but also the success stories brought up during this process. Thus, we are confident that we have gained valuable knowledge in the course of this experience, which can be of great help for similar projects.

## Methods

### Requirement analyses

Before starting the actual development process, the first step was to assess the initial IT situation at the local biobanks in order to identify the existing implementation gaps and to determine the demand for the planned IT solutions. This was achieved by consulting all biobank IT managers at the participating sites. The biobank IT managers compiled and reported back the relevant information from their site, which was then analyzed by the team. In addition, targeted stakeholder workshops and surveys were held over the course of the project with IT experts, biobank managers, scientists and patient representatives in order to gain a holistic view. In accordance with the evaluation of these activities, the requirements were defined, regularly revised and adapted to the actual needs of the biobanks and stakeholders.

### Mode of operation

**Fig 1:**
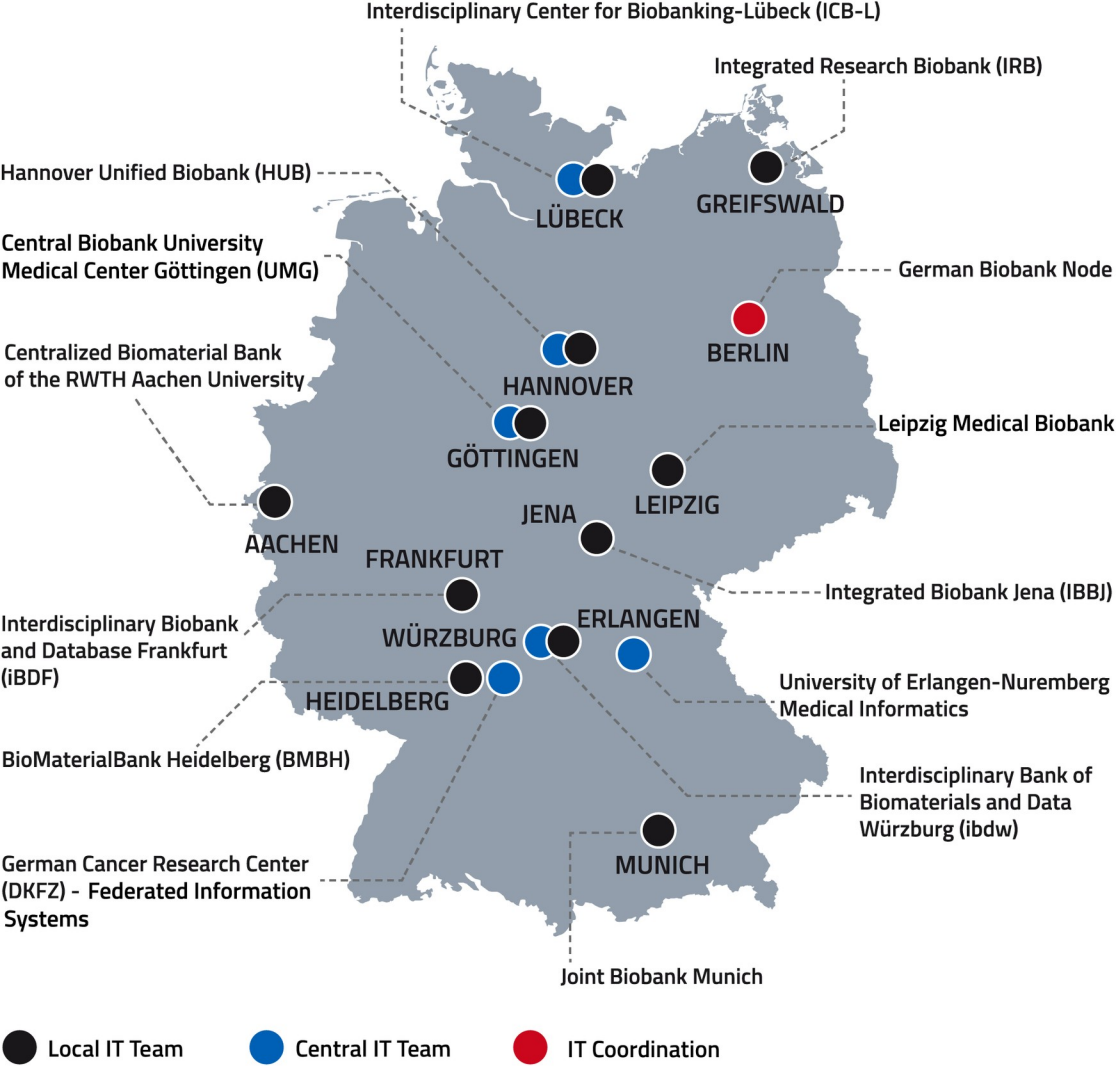
Overview of the partners sites including biobanks, IT teams and GBN at project launch 2017. Printed under a CC BY license, with permission from the German Biobank Node, original copyright 2017.

Two teams were assembled to handle the required IT tasks. One team, consisting of 9 members across 6 partner sites, was responsible for development and maintenance of the IT solutions. The second team, consisting of one IT employee per biobank location, took care of the local integration of the distributed IT components including their connection to the internal data source systems and day-to-day operation. According to the agreement of the project partners, the teams had to be organized to cooperate efficiently across many locations (Fig 1) and to ensure close communication. Therefore, regular conference calls and face-to-face meetings within and between the two teams took place. Additionally, a chat platform was employed for asynchronous exchange. The development team oriented itself towards the agile scrum framework [10]. This was realized by doing sprints in 2–4 weekly intervals, with planning and review taking place at the beginning and end. In addition, short daily web conferences were held for tight coordination. This approach supported the team in working iteratively and responding quickly to changes in requirements.

### Development approach

A strategic idea at the beginning of the project was to build on already existing IT solutions developed in previous projects of the developers from GBA. This refers in particular to the development of the local components. For these, the comprehensive “bridgehead” architecture of the German Cancer Consortium (Deutsches Konsortium für Translationale Krebsforschung [DKTK]) was used as a central element [11]. With regard to the technologies to be used to further develop the existing components, those already established in DKTK were initially retained for GBA in order to keep the migration effort low. Using an iterative approach, the source code was gradually refactored to ensure maintainability and to consider modern architectural patterns. Other components and their libraries had to be replaced completely. However, the knowledge gained by using legacy components largely supported the implementation of the replacing source code. Vice versa, enhancements were created in a manner allowing them to be backported to the DKTK implementation. For example, the installation process of the software had been modernized by GBA and could be used by DKTK.

### Evaluation of components

In the course of the iterative approach ensuring that the developed components are ultimately used according to their purpose, systematic evaluations were carried out. Two usability analyses were conducted to evaluate the user-friendliness of the feasibility tool. The first one was based on a prototype of the user interface in the development phase, while the second one was carried out in a comparative analysis of three query builders in productive use. For both, user-friendliness was assessed using the widely used and standardized System Usability Scale [12] and qualitative questionnaires [13, 14]. Moreover, the functionality of an additionally connected donor portal was evaluated by providing the biobanks with a demonstrator, which was then assessed using an exemplary user scenario and an associated checklist.

### Coordination with other initiatives

In all efforts with focus on national biobanking, attention was paid to ensuring cooperation and coordination with similar initiatives at national as well as international level to establish interoperability and prevent parallel structures. Therefore, a close cooperation with BBMRI-ERIC, as European biobanking organization, took place. The cooperation was carried out within the context of joint working groups. The same principle was followed when working with initiatives that emerged during this project and pursued similar goals. This applies in particular to the German medical informatics initiative (MII), which aims to enhance the secondary use of data generated in the course of patient care [15].

### Ethics statement

Approval was obtained from the responsible ethics committee before the start of each evaluation study. For the first study of the Charité –Universitätsmedizin Berlin [EA1/098/19] and for the second study of the Technical University of Dresden (Germany) was obtained [SR-EK-262062020]. The prerequisite for participation in the studies was written informed consent from the participants in both cases. In addition, written consent was always obtained from external persons if they participated in workshops as part of the project. In the case of surveys conducted among GBA project partners, consent was waived.

## Results

The following section describes the IT specific content of the GBA project.

### Prerequisites at the sites

The analysis of the local IT situation confirmed that some areas had not yet been covered by all participating biobanks, in particular consent management (missing in 6 out of 12 biobanks), project management (4/12), contact management (11/12) and donor empowerment (10/12). Furthermore, it was necessary to elaborate a central data protection concept concerning processes and components for the cross-linkage of biobanks that complements existing local data protection approvals. However, there was no need for a central identity (ID) management and pseudonymization service, since all GBA biobanks already maintained a system compliant to the respective requirements. A further insight from the gap analysis was the use of different IT tools in biobanks. Therefore, we aimed at a high degree of interoperability, clear definition of interfaces and standard IT techniques.

### IT infrastructure

**Fig 2:**
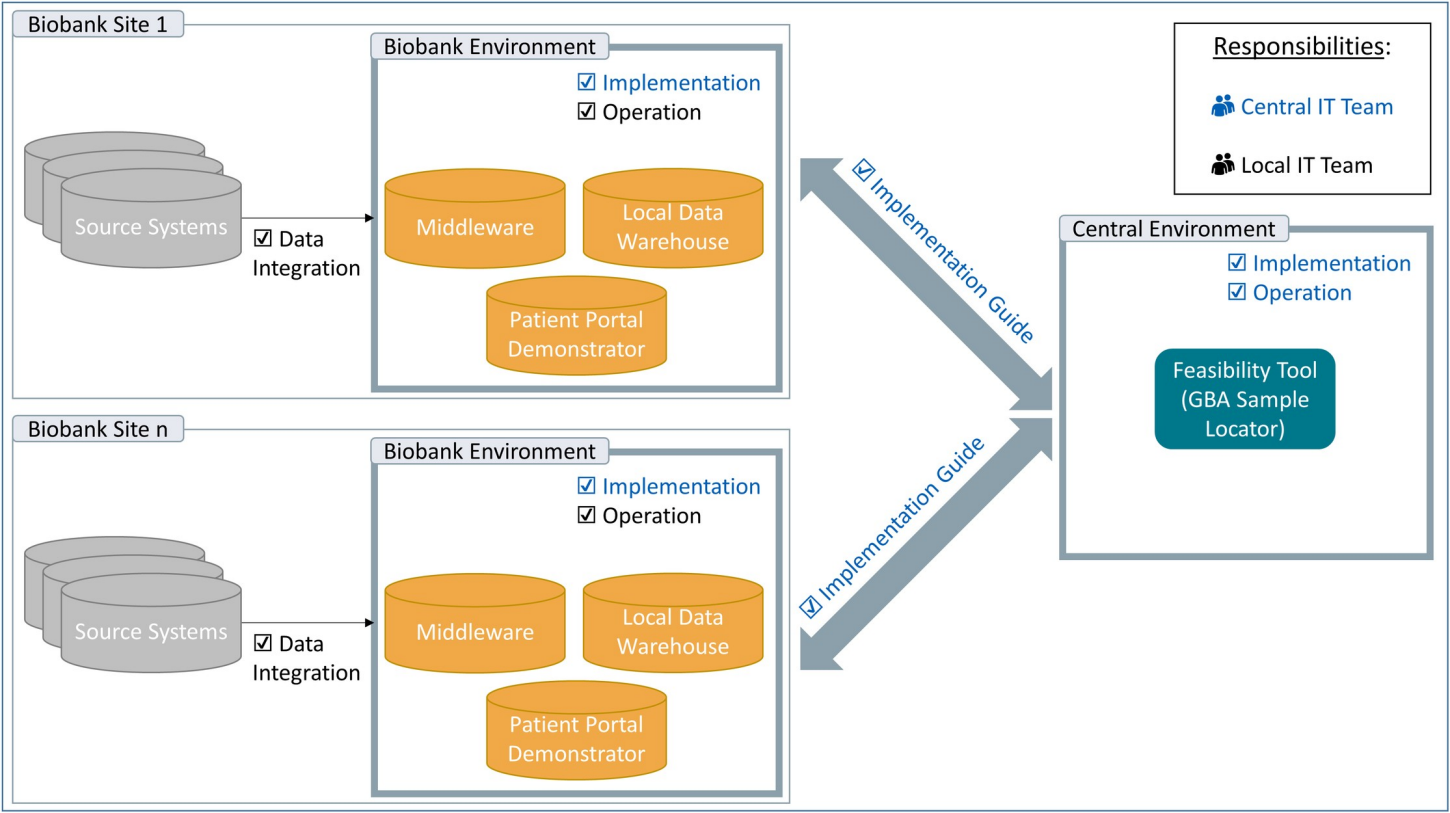
Overview of the IT infrastructure and distribution of responsibilities among the IT teams.

The IT support for biosample and data search is built on a trinity. This comprises (1) the Sample Locator as graphical user interface (GUI) of a central feasibility query, (2) the local data warehouse (DWH), and (3) the middleware as a link between biobanks and the query tool (compare Fig 2).

The first point of contact for researchers looking for biosamples is the Sample Locator as a web-based application for feasibility queries [16]. This enables immediate feedback regarding the number of biosamples or donors for research projects potentially available in the biobanks. A special feature here is the two-stage process that emerged as a requirement from the stakeholder survey of biobank managers. This process involves a first step providing a cumulative highly aggregated number of all biosamples fulfilling the search criteria that can be identified across the biobanks in real-time without authentication. In the second step, registered and authenticated requesters receive a breakdown of the aggregated biosample numbers across the respective biobanks [17]. To this end, the biobanks have to install two local components into their infrastructure specifically developed for this purpose. The DWH stores the necessary data after harmonizing by means of an ETL (extract, transform, load) process from heterogeneous sample and clinical data sources using a consolidated data set. At this point, GBA could make use of existing terminologies and standards, such as MIABIS (Minimum Information About Biobank data Sharing) [18] and SPREC (Standard PREanalytical Code) [19]. Based on these, a corresponding core data set for donor (basic clinical) data, sample data, and disease specific (oncological and cardiological) data was developed in several rounds of balloting and agreed upon in close collaboration with all GBA biobanks.

The middleware serves as an interface between DWH and the feasibility tool and transmits the relevant information according to the request that was submitted. As this connector component is used in several projects, a substantial work of coordination was necessary to adopt the component successfully.

The working modus allowed flexible design decisions even during the project. For instance, for the local DWH, we initially started with the DKTK DWH data model. However, after careful examination, it turned out that its performance was too slow to allow real-time queries and furthermore, the proprietary data format was not interoperable. Moreover, not all research requests based on biobank inquiries could be answered satisfactorily by the underlying query language. This insight resulted from an analysis of concrete search queries we collected from researchers and biobanks, which we then validated against our current development status. Since HL7®FHIR® (Health Level Seven Fast Healthcare Interoperability Resources) [20] gained impact as a health care data transfer standard during the course of the project, the first attempt to solve this issue resulted in the switch to the FHIR® standard query mechanism (FHIR® Search). This way we could overcome the lack of interoperability. However, FHIR® is more intended as an exchange format and not necessarily for querying. Screening of alternatives led to the decision to implement the Clinical Quality Language (CQL) [21]. With CQL, the backend can answer all queries generated by the Sample Locator. The implementation started early in 2019 by defining biobank and biosample specific profiles based on the previously consolidated data set and publishing them on the open platform for HL7® profiles [22].

Successful query results delivered from the Sample Locator need to be communicated to the respective biobanks for further refinement and finally to access to biosamples and data. This is realized by an already developed IT-component, the BBMRI-ERIC Negotiator [23]. By connecting this communication platform to the Sample Locator, any researcher, who has registered via the integrated authentication service BBMRI-ERIC AAI (Authentication and Authorization Infrastructure), may initiate further communication with the respective biobanks [24].

To round up the data processes with and within the biobanks, the contact and consent management with the sample donors needed to be considered as well. During a workshop with patient representatives on this topic, it became evident that patients would not necessarily contact the biobank but rather the clinical institution where the patient was treated and where the biosample was taken. Based on this insight, a component for donor empowerment needs to be embedded into the clinical environment, e.g., as part of an electronic health record (EHR)-integrated patient portal instead of the biobank. For this reason, we recommend for sustainable implementation to rather focus on industrial EHR-vendor-based developments. However, a demonstrator that includes a patient portal with consent functionality and a consent management database, an ID management tool, a project management tool, and an exemplary research database has already been piloted and can be built upon [25].

A detailed description of the IT infrastructure, its components and the underlying technology is the subject of a separate manuscript, which is prepared parallel to this manuscript.

### Sustainability

To ensure the sustainability of our results, all developments are made available as open source under a general public license, so that interested parties can easily access and benefit from the provided IT components. These are available in a GitHub repository set up for this purpose. Moreover, an open-source community resulting from this project is supposed to keep the published code maintained, modified and updated beyond the end of the project. This community is open to everyone and should grow steadily in its further existence. A corresponding declaration was formulated for this purpose [26].

### Local implementation

After all legal requirements had been accomplished and the IT components were deployed, the final step was to set up and operate the infrastructure on site in the respective biobanks by the members of the local IT team. This step included on the one hand the integration of the IT components into the biobank infrastructure and on the other hand the provision of the biobank data via the connection to the required source systems. This was realized via an ETL route specifically developed at each biobank which translates the source data into the specified data format. This phase was particularly characterized by close cooperation between the two IT teams. There was constant consultation and several iterations to adapt the implementation and ETL to local conditions. Once this connection was successfully established using a designated implementation guide [27], the respective biobank with its sample count was visible in the Sample Locator for a researcher’s request for biospecimens (see also Fig 2).

### Evaluation of components

The first usability analysis of the Sample Locator’s graphical user interface was based on a mock-up and came to the conclusion that potential end users found the tool to be intuitive and easy to handle [13]. A further usability analysis based on the productive system could confirm this impression [14]. Nonetheless, both analyses revealed useful potential for improvement that could be incorporated into the subsequent development iterations.

By contrast, the evaluation of the patient portal demonstrator focused on the technical feasibility of integrating it into the biobank infrastructure. By means of a checklist, the functionality of the system was checked after the installation. The review of the completed checklists showed that the implementation of such a portal is in principle technically possible. This finding is an important input to increase the acceptance for the integration of such a portal into a hospital infrastructure.

## Discussion

The principal objective of our work within this project was to establish a functioning IT infrastructure for networking biobanks, an endeavor that can be regarded as successfully accomplished. Nevertheless, several aspects arose in the course of the project that, in retrospect, deserve special mention and attention (Table 1).

**Table 1:** Short summary of challenges, lessons learned, and success stories we encountered during the GBA project.

Challenges
• IT development across locations with heterogeneous background
• Coordination with other research infrastructures: communication and technical interoperability
• Clinic’s patient portal for consent management instead of a biobank specific donor portal
Lessons Learned
• IT development across locations with specific human resources
• Pursuit of open source development
• Comprehensive documentation & testing
• Alignment of the development with real world needs
• Continuous real-life monitoring (technically and manually) of the entire system
• Strong stakeholder involvement, e.g., user centered design approach and usability evaluations
Success Stories
• Early adaptation of HL7 FHIR to ensure interoperability of the biobank’s system
• Containerized deployment & detailed implementation guide
• Agile system development, e.g., enabled to offer specific access to COVID-19 samples online as early as April 2020

### Challenges

Federated IT developments across locations turned out to be a challenge of its own [28]. The spread of the teams across different sites, especially the development team, initially required a certain orientation phase before beginning the actual work on the project. This proved to be necessary since an efficient mode of operation had to be established first. After hiring of staff, an agile development approach with daily scrum meetings and sprints was defined. Tools for project management and a platform to document the progress of work supported this method. In addition to this regular interaction, a chat tool has also been successfully used as a means of asynchronous communication. When setting up the working environment, it is particularly important to pay attention to the regulatory framework of the individual biobank/hospital locations, as certain software, e.g., Zoom for video conferencing or Docker for container virtualization, may not be permitted. Moreover, it should be ensured that all team members have access to the source code and the corresponding development environment. This can be a bottleneck especially if it is hosted at one of the sites and managed only by this site’s local admins. Another aspect that can quickly be neglected in a distributed workspace is the communication and transparency of decisions. In our specific case, this was aggravated by distribution of the project responsibility among two of the central IT sites. Consequently, some changes in the course of the project or with regard to the prioritization of work packages were passed on with some delay.

A challenge that has arisen from external circumstances was and still is the coordination with other research infrastructures, in particular the European BBMRI-ERIC and the German MII. Due to partially overlapping goals within different settings, this reconcilement was essential to avoid the development of parallel structures. With regard to BBMRI-ERIC, significant synergies could be created. The cooperation in terms of AAI [24], the Negotiator [23], and the dataset based on MIABIS [18] led to a rather quick technical integration of the tools within our IT infrastructure and resulted in international visibility and a high degree of interoperability. However, one limitation of this adoption, which should be kept in mind, is the possible limitation in functionality as in the case of the Negotiator as project management tool. At the moment, it is limited to a chat-like communication between biobank and researchers in contrast to the targeted project proposal management tool.

On the other hand, such cooperation can also lead to the components being raised to another level, as in the example of the donor portal, which is now being continued at hospital level with a new funding call (Digitale FortschrittsHubs Gesundheit; digital progress hubs) of the MII [29]. Another field concerns the coordination of the definition and integration of data sets to ensure interoperability of the collected data. Towards this goal, the MII is especially active in developing a standardized core data set for data integration centers since 2019 and e.g., the definition of the biosample data module was therefore closely coordinated with the respective MII initiative. Thus, we have been successful to interact with most relevant task forces to contribute with our expertise, especially in the field of biobanking. However, it is still crucial to make work and developments among the activities transparent and to communicate with each other.

### Lessons learned

Recruiting personnel for a project of this dimension is crucial to reach the full potential. Especially for those locations with a need for new recruitments there is an obvious delay, whereas locations that can build on already available experts are in a better situation. Furthermore, there was initially no coordination planed between the locations of the IT development team regarding the competencies that would have been needed for this project. Particular attention should be paid here to ensuring that certain areas are covered. Within this project, it concerned the following fields: system administration, back-end and front-end development, user interface design and operations. In addition, a person with experience in project management, who has an overview of all work packages, is of utmost importance. Due to the partial lack of coverage of the various expertise, additional support from some biobanks was required. Before starting a project, we suggest considering to what extent the designated team members or temporary interns can or could contribute to the tasks at hand.

A further difficulty was the partial fluctuation of employees almost from the beginning. In particular, the small number of developers amongst the IT development team suffered by friction losses. The attempt to apply pair programming partially healed the situation. However, some expertise was lost and the synchronization as a team was distracted. The changes in staff also affected the rest of the team including the management level and thereby put a strain on the project’s proceeding. However, the motivation within the team helped to overcome this obstacle, even attracting IT developers from outside the original IT team.

In addition to human resources, the availability of infrastructure can also become a bottleneck. Here it should be ensured that a stable environment is provided. Of particular importance is a stable server infrastructure that is permanently available in terms of employee access rights and performance. After initially using a server hosted by one of the IT competence centers for the project, we eventually switched to a cloud server. This enabled us to become independent of location-immanent restrictions with regard to admin rights and ensures continued maintenance. With this approach, though, sufficient funds should be earmarked for the maintenance of such an external service.

The postulate of sustainability, especially the publication of the software code as open-source that was a rightful claim of the funding body and has been recognized as essential, turned out to be rather time and resource consuming. This was mainly due to the fact that work within this project was partially based on already existing software components. For open source publication, there are different license types that can be used. Here, a decision was required as to which of these types should be used for any further publication. The clarification required legal advice and a consensus process between the project team and the legal representatives of the various universities involved. The effort and time required for such a process were unfortunately underestimated at the start of the project. To this end, we strongly recommend that all relevant legal topics should be as far as possible identified and consented already by the start of a project. If existing code cannot be published as open source in the first months one should consider alternatives–e.g., we have developed both the store and the GUI of the Sample Locator from scratch and could thus easily publish them as open source.

In principle, it is favorable to use existing code in order to achieve higher productivity. However, the development speed highly depends on the chosen code base, which should satisfy common standards such as an up-to-date architecture, clean code, and reliable automated tests. For example, adopting a hard to read software that is insufficiently covered by automated tests extends the risk of errors—thereby imposing unnecessary workload on the team—and slows down developers while writing and reviewing code. Moreover, a monolithic structure that is used by more than one development team demands for proactive coordination. Hence, analyzing the code base right at the beginning and being open to switch to another code base or even start from scratch can drastically increase the overall performance of the development team.

In IT-development, it is most crucial to stay connected with the requirements from actual use cases and the stakeholders, especially the users of the proposed tools. Since our project was not aiming merely at connecting biobanks across different locations, but also to enable specific queries for biosamples to fuel scientific work, this needed to be outlined thoroughly. For this purpose, we collected “real world” sample requests to test the system and realized that these requests were by far more complex than anticipated. This was complicated by false positive results that disclosed discrepancies as the donor’s age and the sample-derived diagnosis not matching though both were entered for the search. This was detected by constant development and technical as well as manual testing. This “real-life” monitoring definitely can be seen as one valuable lesson learned.

Another aspect that this “real-life” monitoring revealed was that both frontend design and user-friendliness had been underestimated within the project plan. The complexity of the “simple” sample requests, gathered to test the system, was not easily covered by building a generic user interface. Though the backend using CQL imposed no restrictions for the “real-life” queries defined by researchers, the generic approach to configure the frontend using a metadata repository and implementing simple logic on fields, values, and operators failed in this regard. To make up for this omission, the team took part in a usability workshop and applied the acquired knowledge in a subsequent evaluation of a sample locator prototype [13], the results of which could be used in further development. Another lesson learned regarding the graphical interface concerned the framework. The first attempt was built on the existing code using Java Server Faces which was hard to maintain and almost impossible to enhance with new features. We therefore decided to switch to a proper web framework (Angular) because it is commonly used and suitable also for developers who are not specialized in web development. In order not to neglect the cooperate design of the project, the GBA’s public relation coordinator supported the migration and development. Considering this, we recommend to not underestimate the user’s experience in academic IT-developments since user interfaces that are intuitive and well suited for the end user increase the acceptance of the tools and thereby ensure the success of a project beyond its end [30].

### Success stories

One major success of this project was the early adaptation of the HL7®FHIR® standard to enhance interoperability. Originally, the HL7 standard was developed, among other things, for the communication of patient data within a hospital and has become widely established. For intersectoral communication, on the other hand, the relatively new FHIR standard was developed, so it was not yet widely used in academic research, at least in the biobanking community. However, since this community operates at the interface between patient care and biomedical research, we decided to adopt it for our purpose, namely saving and querying biobank data. Accordingly, we have translated the metadata of the sample, donor, and biobank data set into so-called FHIR profiles [22]. With those, the relevant data can now be easily standardized within the established IT-infrastructure to make them easier to query. Although the decision to follow this path was made relatively late in the project, it was consistently pursued by the cooperation of the team by swiftly creating profiles of the biobank data set approved by the community. The smooth implementation of this relatively new standard in biobanks was favored by several factors: 1) no established format for cross-site communication in the biobank community, so FHIR was able to fill this gap without displacing existing standards while adding value to the given biobanking standards MIABIS and SPREC that were consulted and respected during profiling, 2) the commitment of the project partners to introduce this largely unfamiliar standard locally in the short term, 3) the general developments within the MII, in which all German university hospitals also agreed to use the FHIR standard, and 4) the fact that FHIR is structurally based on established HL7 standards, namely version 2 and CDA (Clinical Document Architecture), and that the aim is to harmonize FHIR and CDA, making mapping straightforward. As part of the decision to switch to FHIR, we also developed and delivered a completely new local DWH, the Blaze Store [31], within a short period of time. Although this meant additional effort, it also brought the advantage that the new store could be tailored for the FHIR standard. This not only represents an additional benefit for GBA, but also is currently being considered for use in other projects, including MII.

The activities of GBA also attracted the interest of other biobanks, which eventually applied to join the alliance as partners [32]. Provided they met a predefined minimum requirement [33], they were accepted as active members of the network. With regard to the delivery of the IT tools, this posed a particular challenge since not all biobanks have their own IT staff. It was therefore all the more important to offer an approach that makes it as straightforward as possible for biobanks to deploy the required tools. The decisive factor to achieve this was the decision to deliver the tools in a containerized version and to publish a detailed implementation guide [27, 34]. Furthermore, the new members benefited from the experience of the trained IT staff, who were able to pass on approaches to solving emerging problems expeditiously via the established communication channels. Like this, 14 biobanks could already be connected to the Sample Locator in a short time.

**Fig 3:**
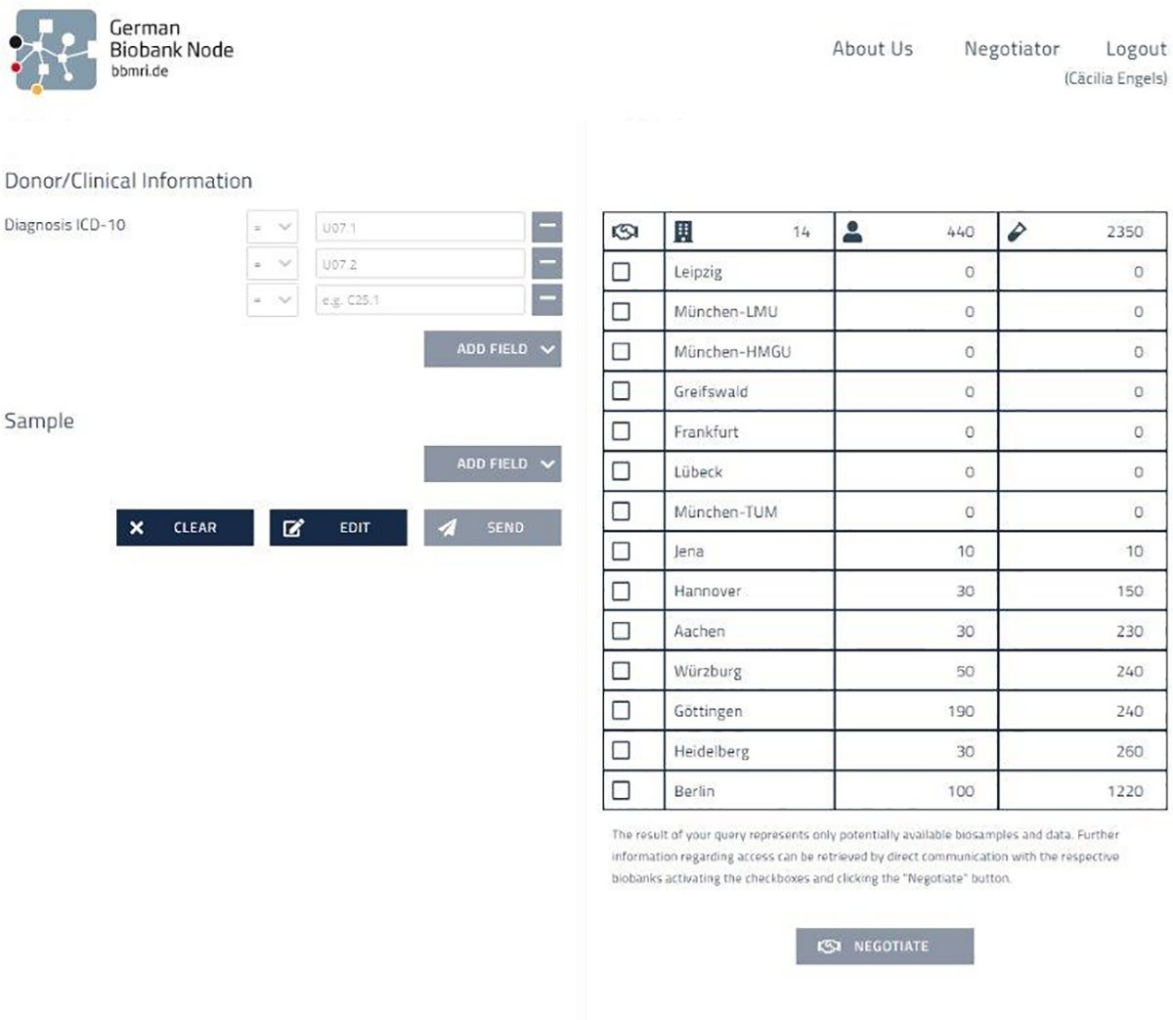
Samples available in the Sample Locator as early as 28^th^ of May 2020 from donors diagnosed with U07.1 or U07.2 (COVID-19).

Effective dissemination of knowledge during the development of the IT infrastructure allowed to quickly react to recent developments. For example, only just released for productive use at the end of 2019, the IT infrastructure of German biobanks had to withstand a first baptism of fire in 2020 with the outbreak of the COVID-19 pandemic and the soaring demand of researchers for high-quality SARS-CoV-2 biosamples. Once the collection and storage of varieties of these specific samples from biobanks had begun, the samples could be found immediately online by searching for the International Classification of Diseases (ICD) 10 codes U07.1 (COVID-19) and U07.2 (suspected COVID-19) within the Sample Locator throughout Germany (Fig 3). The German Biobank Node collected requests for SARS-CoV-2 specific samples (and other samples) to track efficacy of the Sample Locator and Negotiator. From launch until today 7 SARS-Cov-2-specific sample requests for different sample types with or specifically without mentioned diagnosis have been received of which 4 requests could be mediated successfully.

## Conclusion

The use of high quality biosamples and their associated clinical data from biobanks are essential to produce successful, reliable and reproducible biomedical research data. To meet this demand, we have started to create an IT network of German biobanks that enables feasibility queries across all connected sites. This was only possible by the efforts of a federated T developer team which was active on behalf of the German Biobank Node/Alliance. However, collaboration in such a distributed team requires the consideration of several prerequisites that need to be taken into consideration before starting the project. In this paper, we want to share our experience which might be of value for similar endeavors, and which should help to prevent unnecessary mistakes (Table 2).

**Table 2:** Six key recommendations for similar projects.

1. Clarify legal conditions! Issues such as open source licensing should be settled with all affected parties before or early in the project, in written form if necessary.
2. Recruit appropriate staff! Specify what expertise is needed and coordinate with project partners when staffing the project.
3. Designate one permanent team leader! This person keeps track of project milestones and distributes precise tasks according to their urgency to team members with the adequate skill.
4. Pay attention to areas of responsibilities! The team receives work assignments from the team leader, who in turn communicates with the project management. Unclear hierarchies can be misleading.
5. Be flexible, but thoughtful! New conditions/requirements/technologies should be addressed, but not rashly.
6. Communicate! Involve your team in decisions and listen to its thoughts.

In fact, we are already able to benefit from our success and experience gained in this GBN/GBA project for the biobank IT activities within the ABIDE_MI (Aligning Biobanking and DIC Efficiently) project [35]. This is a successor project that has emerged from the previous cooperation between MII and GBN and even includes more biobank sites.

## Abbreviations

AAI: Authentication and Authorization Infrastructure
ABIDE_MI: Aligning Biobanking and DIC Efficiently
BBMRI-ERIC: Biobanking and Biomolecular Resources Research Infrastructure–European Research Infrastructure Consortium
BMBF: Bundesministerium für Bildung und Forschung (Federal Ministry of Education and Research)
CDA: Clinical Document Architectur
CQL: Clinical Quality Language
DKTK: Deutsches Konsortium für Translationale Krebsforschung (German Cancer Consortium)
DWH: Data Warehouse
EHR: electronic health record
ETL: extract, transform, load
FHIR: Fast Healthcare Interoperability Resources
GBA: German Biobank Alliance
GBN: German Biobank Node
GUI: graphical user interface
HL7: Health Level 7
ICD: International Classification of Diseases
ID: identity
IT: information technology
MIABIS: Minimum Information About Biobank data Sharing
MII: Medical Informatics Initiative
SPREC: Standard Preanalytical Code
